# The Aesthetic Developmental Characteristics of Contour Features in Children and Adolescents with High- and Low- Level Visual Aesthetic Sensitivity across Grade Levels

**DOI:** 10.3390/bs14050416

**Published:** 2024-05-15

**Authors:** Ju Zhang, Zijia Lu, Yongsheng Wang, Xuejun Bai

**Affiliations:** 1 Faculty of Psychology, Tianjin Normal University, Tianjin 300387, China; orange686@163.com (J.Z.); wangyongsheng@tjnu.edu.cn (Y.W.); 2 Department of Applied Psychology, Law School, Southwest University of Science and Technology, Mianyang 621010, China; 3 Law School, Tianjin University of Commerce, Tianjin 300131, China; luzijia_psy@163.com

**Keywords:** contour features, children, adolescents, high and low levels, visual aesthetic sensitivity, developmental characteristics

## Abstract

This study examined the aesthetic developmental characteristics of contour features (curved and sharp corners) among children and adolescents with different levels (high and low) of visual aesthetic sensitivity in three grades (4, 6, and 8). The results revealed that (1) there was a significant main effect of contour features, with children and adolescents liking curved contours and perceiving them as more beautiful than sharp-angled contours; (2) there was a significant interaction with contour features in grades 6 and 4, and there was no significant difference in liking curved contours and perceiving them to be more beautiful between students in grades 6 and 4. However, grade 6 students disliked sharp-angled contours and perceived them as more unattractive than grade 4 students; and (3) there was a significant interaction between the level of visual aesthetic sensitivity and contour features, as children and adolescents with both high and low levels of visual aesthetic sensitivity preferred curved contours and considered them more beautiful. However, children and adolescents with high-level visual aesthetic sensitivity disliked sharp-angled contours and considered them more unattractive compared to students with low-level visual aesthetic sensitivity. The results proposed that children and adolescents preferred curved contours, 6th graders were more sensitive to curved contours than 4th graders, and children and adolescents with high-level visual aesthetic sensitivity were more sensitive to sharp-angled contours than children and adolescents with low-level visual aesthetic sensitivity.

## 1. Introduction

Contour is an essential visual feature of object shape, a critical visual cue for searching and recognizing objects, and plays an important role in visual aesthetic evaluation [1]. As an important source of information for visual perception, different contours and the lines that compose them give different feelings [1,2,3]. Curved lines give a feeling of tenderness, quietness, and gentleness and sharp lines give a feeling of agitation, hardness, and anger [4,5,6]. Contours are categorized into two types: curved contours and sharp-angled contours. Curved contours have curvature that varies smoothly along the contour, while sharp-angled contours vary abruptly along the contour (curvature discontinuity) [7]. The study of aesthetic preferences has been a topic of psychological research [8], with much focus on identifying the perceptual features that drive them [9]. Previous studies have found that people prefer objects with curved contours over those with sharp angles, even with similar shapes [3,10,11,12,13,14]. The preference for curved contours is a widely observed phenomenon that has been confirmed in various stimuli, such as meaningless patterns [7,10,12,15,16,17,18], meaningful patterns [9,11,19,20,21], typefaces [22,23], paintings [24], car interior designs [25], product designs [26], furniture [27], and architectural and interior environments [18,28,29,30]. The aesthetic preference for curved contours has also been confirmed in different cultures [1,2,21,31] and species [9]. There are two primary approaches to the study of contour features. The first approach involves presenting two shapes that are similar except for their contours and then asking participants to choose between them [9,10,16]. The second approach involves showing only one curved or sharp-angled contour pattern at a time and asking participants to rate it based on how much they like it or how beautiful it looks [7,12,15,18,19,20,32]. Various factors influence preference for curved contours, such as stimulus presentation time [9,16], gender and major [17], moderation of artistic expertise [3,12,30], the aesthetic preferences of the times [33], learning, and the mere-exposure effect [29].

Newborns and infants prefer to gaze at or choose curved contours objects. Studies have found that newborns [34] and 3-month-old infants [35] spend significantly more time gazing at curved contours than straight contours; 3- to 4-month-old infants prefer curved contours [36], and curves appear to have a stronger attraction to 10-month-old infants than straight lines [37]. Jadva et al. [38] found that 3-year-olds preferred curved contour toys over angular ones. Do children and adolescents also prefer curved contours? It is surprising to note that older students are not given enough attention as participants in research studies, despite the growing number of research studies being conducted. Their valuable insights and experiences have yet to be fully explored, and it is important to recognize the significance of including them in research studies. Therefore, this study used children and adolescents as participants to answer this question. Based on the existing studies, we hypothesize that children and adolescents in different grades also prefer curved contours regarding contour features.

There are general differences between individuals with high and low levels of visual aesthetic sensitivity. Aesthetic sensitivity refers to the extent to which an individual responds to aesthetic stimuli based on consistency and appropriateness with external standards [39]. Visual aesthetic sensitivity is a universal, objective visual aesthetic factor in aesthetic appreciation [40,41] that captures differences in individual aesthetic abilities [42,43,44], with differences primarily in the aesthetic quality of the recognition [45,46] and judgments of artwork quality [47]. Individuals will demonstrate a higher ability to identify aesthetic quality if they can identify, analyze, and assess deficits in various aesthetic features on a visual aesthetic sensitivity test [48]. The Visual Aesthetic Sensitivity Test (VAST) was developed by Götz et al. [42] and later revised [49]. It has been widely used to examine visual aesthetic sensitivity [41,46,50,51,52,53]. Historically, there have been other visual aesthetic sensitivity tests, such as the Maitland Graves Design Judgment Test [54] and the Meier Art Tests [55]. The VAST has favorable psychometric properties [56], making it the only recommended test for visual aesthetic sensitivity [57]. Child [58] found general differences between individuals who scored high on a visual aesthetic sensitivity test and those who scored low on a visual aesthetic sensitivity test, both in elementary school and middle school. Individuals with high levels of visual aesthetic sensitivity pay more attention to higher-order features and the overall structure of the aesthetic stimuli, giving higher-than-average weight to the more beautiful aesthetic stimuli [48,59], and individuals with low levels of visual aesthetic sensitivity pay more attention to the individual objects that are directly available in the aesthetic stimuli [48]. Contours are generally considered more aesthetically beautiful and pleasant than sharp-angled contours [7]. Therefore, it is hypothesized that students with high levels of visual aesthetic sensitivity would prefer curved contours over sharp-angled contours. On the other hand, it is assumed that students with low levels of visual aesthetic sensitivity have no significant difference in their liking for the two types of contours.

This study focused on investigating the developmental characteristics of the perception of contour features among students with high and low levels of visual aesthetic sensitivity at different grade levels. The study participants were 4th and 6th grade elementary and 8th grade middle school students. The VAST screened students with high and low levels of visual aesthetic sensitivity, and meaningless curved contour and sharp-angled contour patterns were chosen as stimulus materials. The research hypotheses were as follows: (1) children and adolescents in different grades preferred curved contours in terms of contour features; (2) students with high levels of visual aesthetic sensitivity preferred curved contours, and students with low levels of visual aesthetic sensitivity did not have a significant difference in preference for the two contour features.

## 2. Methods

### 2.1. Participants

**Figure 1:**
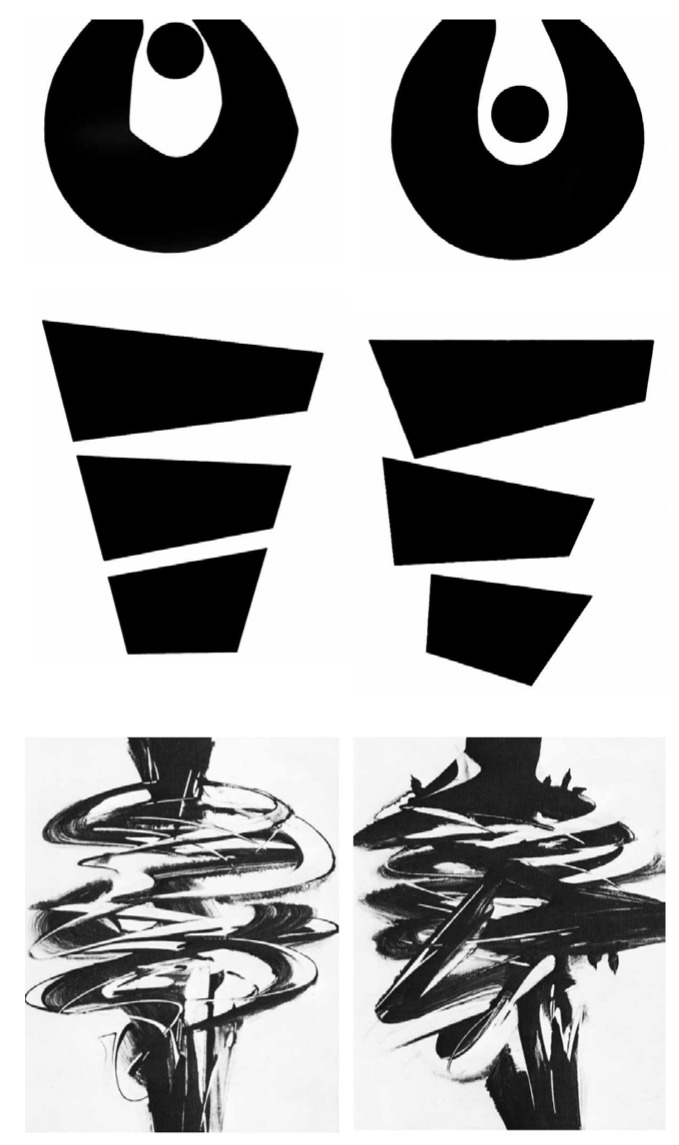
Example items of the Visual Aesthetic Sensitivity Test (from [49]).

A total of 171, 182, and 160 participants were selected from grades 4 and 6 of an elementary school and grade 8 (the X and SD of the actual ages of the participants in each grade can be seen in Table 1) of a middle school in Mianyang, Sichuan Province, respectively. They were collectively administered the VAST to assess their visual aesthetic sensitivity. It has been tested and confirmed that the test is reliable among Chinese students in our other study (internal consistency alpha coefficient of 0.69, retest reliability of 0.74, that manuscript is unpublished in China). The Chinese version of VAST adopts a double-blind translation. Two English major graduate students are invited to translate the original English version of the test independently of each other, and two college English major teachers are invited to translate the Chinese version back into English and compare it with the original. There are 50 items in the VAST (example items of the VAST are presented in Figure 1), and each item consists of a pair of abstract pictures. “Each pair consist of two quite similar pictures one of which is superior from the point of view of design; it is more harmonious, better balanced and better adapted in the way the elements are ordered, and in the way the lines are drawn. Look carefully at the picture, and you will see that in the comparison the worse picture contains small ‘faults’ or ‘errors’ which destroy the balance of the picture” [42,49]. If the subjects choose the better designed picture, they will get 1 point, and 0 points if they choose the other picture that upset the balance. Scores range from 0–50, with higher scores indicating higher levels of visual aesthetic sensitivity.

Based on the participants’ test scores, students who scored in the top 27% and bottom 27% of the scores in each grade level were selected as high- and low-level visual aesthetic sensitivity students [60], and the results of the descriptive statistics for each grade level are shown in Table 1. Analysis of the test scores of the students with high and low levels visual aesthetic sensitivity in each grade level revealed that there was no significant main effect for the grade level, *F* (2, 274) = 0.87, *p* = 0.422, $\eta_{p}^{2}$ = 0.01, but the main effect of the high and low levels of visual aesthetic sensitivity was significant, *F* (1, 274) = 1464.67, *p* < 0.001, $\eta_{p}^{2}$ = 0.84. Students with high levels of visual aesthetic sensitivity scored significantly higher than students with low levels of visual aesthetic sensitivity. The interaction between grades and groups is not significant, *F* (2, 274) = 1.58, *p* = 0.209, $\eta_{p}^{2}$ = 0.01. For each grade level, students with high and low levels of visual aesthetic sensitivity were selected as the final participants for the experimental study. Participants had normal or corrected-to-normal vision. Participants provided written informed consent to take part in the experiment procedure, which the Ethical Committee of Tianjin Normal University approved.

### 2.2. Experimental Design

A mixed experimental design of 3 (grade level: 4, 6, 8) × 2 (visual aesthetic sensitivity level: high, low) × 2 (contour features: curved, sharp-angled) was used. Among them, grade level and visual aesthetic sensitivity level were the between-participants variables, and contour features was the within-participants variable.

### 2.3. Materials

Meaningless contour patterns were chosen as stimuli to avoid influences such as stimulus familiarity and the mere-exposure effect [10]. The stimuli were 84 contour patterns (42 curved contours and 42 sharply-angled contours with similar shapes but different contours) selected from the stimulus set created by Corradi et al. [16], and the gray background of the patterns was changed to white using Photoshop CS6, with all other parameters remaining unchanged. The experiment consisted of 4 practice trials and 80 experimental trials.

### 2.4. Experimental Apparatus

The stimuli were presented in the center of a 19″ computer screen with a screen resolution of 1024 × 768. Each computer was equipped with headphones and had the same computer model, screen size, software, and lighting conditions. The experimental task was presented using E-prime 3.0 software, and all stimuli were black and white patterns on a gray background. Each pattern was 680 × 680 pixels, and participants’ eyes were approximately 45 cm from the screen.

### 2.5. Experimental Procedures

Experiments were conducted in the laboratory for students with high and low levels of visual aesthetic sensitivity at each grade level. Audio and text lab instructions were presented simultaneously. Participants entered the practice trial after understanding the experimental tasks and requirements. After familiarizing themselves with the testing procedure, participants entered the formal experiment. During the formal experiment, each trial started with a red “+” gaze point of 800 ms in the center of the screen, followed by a stimulus presented in the center of the screen. According to the two textual prompts at the bottom of the screen, “how much you like the picture” and “how beautiful you think the picture looks”, participants used the mouse to complete two ratings on a scale of 1–7, with 1 for “I don’t like it at all” or “it doesn’t look beautiful at all”, 7 for “I like it very much” or “it looks very beautiful”. After completing both ratings, participants clicked on the “Next Page” button on the page to proceed to the next page. A gray screen was presented for 1600 ms before the start of the next trial. The order of stimulus presentation was randomized, and each stimulus was presented only once.

## 3. Results

The data were screened before data analysis: (1) The data of participants who failed to complete the experiment with regularity of responses and malfunctioning of the program were deleted. One participant was deleted from the high group of grade 4, four participants from the low group of grade 4, six participants from the low group of grade 6, one participant from the high group of grade 8, and one participant from the low group of grade 8. (2) The data of trials in the valid data that were outside of three standard deviations of the rating of the degree of liking of the pattern and outside of three standard deviations of the rating of the degree of the pattern’s beauty were deleted. In the R language environment [61], using the lme4 [62] and lmerTest [63] data processing packages, the degree of liking of the pictures and the degree of the pictures’ beauty were analyzed in linear mixed-effects models (LMM), respectively.

### 3.1. Preference of Contour Features

The means and standard deviations of the ratings of preference and beauty of contour features by students with high and low levels of visual aesthetic sensitivity at each grade level are shown in Table 2.

**Figure 2:**
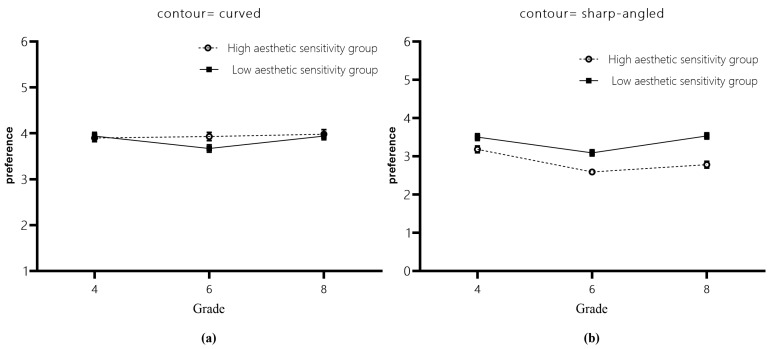
Ratings of preference of contour features by students with high and low levels of visual aesthetic sensitivity. (**a**) The ratings of preference of curved contour feature by students with high and low levels of visual aesthetic sensitivity at each grade level. (**b**) The ratings of preference of sharp-angled contour feature by students with high and low levels of visual aesthetic sensitivity at each grade level.

The main effect of contour features was significant (*b* = −0.78, *SE* = 0.08, *t* = −9.43, *p* < 0.001, 95%CI = [−0.95, −0.62]), with students liking curved contours significantly more than sharp-angled ones. The interaction with contour features was significant for grades 6 and 4 (*b* = −0.37, *SE* = 0.18, *t* = −2.08, *p* = 0.039, 95%CI = [−0.73, −0.02]). A simple effects analyses found that there was no significant difference in the preference of curved contours between grades 6 and 4 students (*t* = −0.60, *p* = 0.822). However, it was found that grade 6 students have a significantly lower liking for sharp-angled contours compared to grade 4 students (*t* = −2.51, *p* = 0.032). Therefore, it can be concluded that grade 6 students have a stronger dislike for sharp-angled contours in comparison to grade 4 students. The interaction between level of visual aesthetic sensitivity and contour features was significant (*b* = 0.61, *SE* = 0.15, *t* = 4.08, *p* < 0.001, 95%CI = [0.32, 0.90]) (Figure 2). A simple effects analysis showed that there was no significant difference in the preference of curved contours between students with high and low levels of visual aesthetic sensitivity (*t* = 0.51, *p* = 0.612) (Figure 2a). However, high-level visual aesthetic sensitivity students liked sharp-angled contours significantly less than low-level visual aesthetic sensitivity students (*t* = −3.18, *p* = 0.002) (Figure 2b), which means that high-level visual aesthetic sensitivity students disliked the sharp-angled contours more than low-level visual aesthetic sensitivity students. The differences in all other effects were not significant.

### 3.2. The Beauty of the Contour Features

The means and standard deviations of students’ ratings of the beauty of contour features at high and low levels of visual aesthetic sensitivity at each grade level are shown in Table 2.

**Figure 3:**
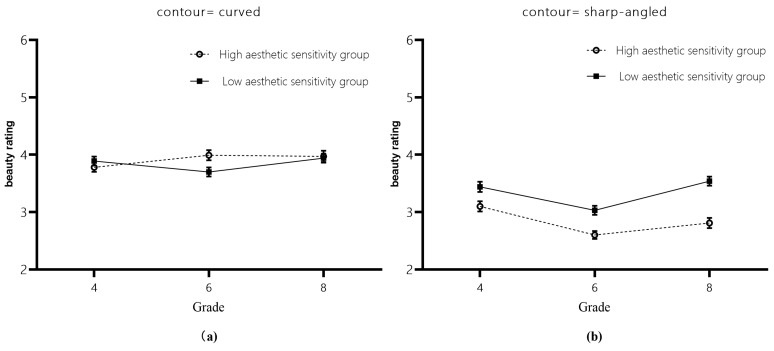
Ratings of beauty of contour features by students with high and low levels of visual aesthetic sensitivity. (**a**) The ratings of beauty of curved contour feature by students with high and low levels of visual aesthetic sensitivity at each grade level. (**b**) The ratings of beauty of sharp-angled contour feature by students with high and low levels of visual aesthetic sensitivity at each grade level.

The results showed that there was a significant main effect for contour features (*b* = −0.79, *SE* = 0.08, *t* = −9.66, *p* < 0.001, 95%CI = [−0.95, −0.63]), with students rating the beauty of curved contours significantly higher than that of sharp-angled contours. The interaction with contour features was significant for grades 6 and 4 *(b* = −0.48, *SE* = 0.18, *t* = −2.68, *p* = 0.008, 95%CI = [−0.83, −0.13]). A simple effects analysis revealed that students in grades 6 and 4 perceived curved contours to be significantly more beautiful than sharp-angled contours ($\left|t\right|s$ > 4.18, *p*s < 0.001). There was no significant difference between the two grades in ratings of the beauty of the curved contours (*t* = 0.09, *p* = 1.00). Still, there was a borderline significant difference in ratings of the beauty of the sharp-angled contours (*t* = −2.29, *p* = 0.058), and 6th graders rated the beauty of the sharp-angled contours lower than 4th graders, meaning that 6th graders perceived the sharp-angled contours as more unattractive compared to 4th graders. The interaction between the level of visual aesthetic sensitivity and contour features was significant *(b* = 0.57, *SE* = 0.15, *t* = 3.91, *p* < 0.001, 95%CI = [0.28, 0.86]) (Figure 3), and a simple effects analysis found that students with both high and low levels of visual aesthetic sensitivity preferred curved contours as more beautiful. The difference in ratings between students with high and low levels of visual aesthetic sensitivity was not significant (*t* = 0.41, *p* = 0.680) when the contour was curved (Figure 3a). However, when the contour was sharp-angled, high-level visual aesthetic sensitivity students rated it as significantly less beautiful than low-level visual aesthetic sensitivity students (*t* = −3.05, *p* = 0.002) (Figure 3b), which means that high-level visual aesthetic sensitivity students perceived the sharp-angled contour as less beautiful compared to low-level visual aesthetic sensitivity students. The differences in all other effects were not significant.

## 4. Discussion

In order to examine the aesthetic developmental characteristics of contour features among students with high and low levels of visual aesthetic sensitivity in different grades, students with high and low levels of visual aesthetic sensitivity were screened from grades 4, 6, and 8 to rate how much they liked the contour and the degree of its beauty, using meaningless contour patterns (curved and sharp-angled) as stimuli. The experimental data were analyzed using linear mixed-effects models, and it was found that there was a significant main effect of contour features, and students preferred curved contours and considered them more beautiful. The interaction with contour features was significant for grades 6 and 4, and there was no significant difference between grades 6 and 4 in their ratings of liking and beauty of curved contours. Still, students in grade 6 disliked sharp-angled contours more than students in grade 4 and considered them less unattractive. The interaction between level of visual aesthetic sensitivity and contour features was significant, with both high and low levels of visual aesthetic sensitivity students preferring curved contours as more beautiful. There was no significant difference in the ratings of liking and beauty of curved contours between high and low levels of visual aesthetic sensitivity among students. However, high-level visual aesthetic sensitivity students significantly disliked sharp-angled contours and perceived them as more unattractive than low-level visual aesthetic sensitivity students.

### 4.1. Visual Aesthetic Preferences for Contour Features

Examining aesthetic preferences for contour features in children and adolescents can help to characterize the aesthetic development of low-level visual features at this stage of life. It was found that students significantly preferred curved contours to sharp-angled contours and considered curved contours to be more beautiful, indicating that curved contours are preferred during childhood and adolescence, which is consistent with our research hypothesis. This suggests that children and adolescents have a stable preference for curved contours. Why do individuals prefer curved contours? It has been suggested that sharp-angled contours convey a greater sense of power [2] and threat [64,65]. Preference for curved contours is due to avoidance of the potential threat triggered by sharp-angled contours [10,66]. Leder et al. [14] found that curved stimuli were preferred over sharp-angled stimuli only when the object had positive or neutral emotional valence, whereas Bertamini et al. [7] argued that preference for curved contours is mainly attributed to the intrinsic characteristics of curved contours, with curves having good continuity [35,36]. Curved contours are inherently more pleasant [7,21,29]. Preference for curved contours is due to convergence to curved contours rather than being based on the rejection of sharp contours [11].

### 4.2. Aesthetic Differences between Grades on Contour Features

It was found that the differences between grades 6 and 4 interacted significantly with contour features. There was no significant difference between grades 6 and 4 students in how much they liked curved contours, or how beautiful they perceived them to be. However, in contrast, 6th graders significantly disliked sharp-angled contours more and perceived them as less beautiful, indicating that 6th graders were more sensitive to sharp-angled contours than 4th graders. This suggests that the dislike of sharp-angled contours fluctuates throughout the children and adolescents’ development. How children engage in aesthetic appreciation and judgment changes as they develop [67]. Aesthetic emotions move toward increased complexity, subtlety, and responsiveness, becoming developmentally more prone to detecting and responding more to certain features and characteristics of artistic stimuli [68,69]. However, aesthetic development is gradual and continuous within and between stages, with possible overlap and irregularity [70]. According to Piaget’s theory of cognitive developmental [71], 6th graders are in a period of transition from the stage of concrete operations to the stage of formal operations. Their way of thinking is shifting from concrete-image thinking to abstract-logical thinking, and the fact that 6th graders disliked the sharp-angled contours more than 4th graders may be related to a shift in how individuals think at this age.

### 4.3. The Effect of Level of Visual Aesthetic Sensitivity on Aesthetic Preference for Contour Features

This study found that students with both high and low levels of visual aesthetic sensitivity considered curved contours as more beautiful, which contradicts the research hypothesis. This suggests that curved contours may be a visual feature that is universally noticed. Students were more likely to see curved contours’ continuity in curvature and evoke a sense of beauty during perceptual analyses. According to this study, students with high-level visual aesthetic sensitivity are more likely to be sensitive to sharp-angled contours compared to students with low-level visual aesthetic sensitivity. They can better distinguish and evaluate the differences between curved contours and sharp-angled contours and tend to rate sharp-angled contours lower in aesthetics. From this study, it can be found that the results of aesthetic preferences for contour features by students with high and low levels of visual aesthetic sensitivity were similar to those of aesthetic preferences for contour features of 6th and 4th graders. Research conducted by Child [58] revealed that students with high-level visual aesthetic sensitivity developed faster than those with low-level sensitivity. Additionally, these students exhibited differences similar to those between younger and older students. These findings are consistent with the current results. Both of these studies suggest that visual aesthetic sensitivity to contour features may vary with grade level, similarly to students with high and low levels of visual aesthetic sensitivity.

### 4.4. Limitations and Future Directions

This study has discovered some interesting findings, but it also has some limitations. First, in addition to the two primary approaches in the study of contour features mentioned in this paper, previous studies using fMRI techniques have found that people differ in the degree of involvement of the orbito-frontal cortex when viewing beautiful and ugly paintings [72,73]; therefore, when measuring an individual’s liking of contour features and how beautiful they look, it is also possible to measure the extent to which curved and sharp-angled contours activate the reward centers, such as the orbito-frontal cortex. Secondly, the use of behavioral experiments in this study did not allow for an investigation into why children and adolescents prefer curved contours, why 6th graders disliked sharp-angled contours more than fourth graders, and the reasons why high-level visual aesthetic sensitivity students disliked sharp-angled contours compared to low-level visual aesthetic sensitivity students. These reasons could be further explored in future studies with a variety of research instruments.

## 5. Conclusions

Children and adolescents preferred curved contours. In comparison to 4th graders, 6th graders were more sensitive to curved contours, and high-level visual aesthetic sensitivity students were more sensitive to sharp-angled contours than low-level visual aesthetic sensitivity students.

## Figures and Tables

**Table 1:** Mean age and test scores of students with high and low levels of visual aesthetic sensitivity by grade level (Standard deviations in parentheses).

Grade	High Level of Visual Aesthetic Sensitivity Students			Low Level of Visual Aesthetic Sensitivity Students		
	n	Age	Score	n	Age	Score
4	46	10.2 (0.5)	37.72 (1.97)	46	10.2 (0.4)	28.00 (1.56)
6	50	12.1 (0.4)	38.34 (2.07)	50	12.2 (0.5)	28.04 (2.99)
8	44	14.0 (0.5)	38.73 (1.74)	44	14.2 (0.4)	27.82 (2.75)

**Table 2:** Means and standard deviations of ratings of preference and beauty of contour features by students with high and low levels of visual aesthetic sensitivity by grade level.

	Curved Contour						Sharp-Angled Contour					
	High Level of Visual Aesthetic Sensitivity			Low Level of Visual Aesthetic Sensitivity			High Level of Visual Aesthetic Sensitivity			Low Level of Visual Aesthetic Sensitivity		
	Grade 4	Grade 6	Grade 8	Grade 4	Grade 6	Grade 8	Grade 4	Grade 6	Grade 8	Grade 4	Grade 6	Grade 8
preference	3.90 (1.31)	3.93 (1.50)	3.98 (1.49)	3.94 (1.43)	3.67 (1.30)	3.94 (1.37)	3.18 (1.37)	2.59 (1.07)	2.78 (1.35)	3.50 (1.42)	3.09 (1.40)	3.53 (1.39)
beauty	3.77 (1.30)	3.99 (1.45)	3.97 (1.58)	3.89 (1.39)	3.70 (1.29)	3.94 (1.35)	3.10 (1.43)	2.60 (1.08)	2.81 (1.44)	3.44 (1.39)	3.03 (1.35)	3.54 (1.36)

Note: Standard deviations are in parentheses.

## Data Availability

The datasets generated and/or analyzed during the current studies are not publicly available but are available from making a request to the corresponding author.
